# Evaluation of a Problem-Based Learning Program’s Effect on Artificial Intelligence Ethics Among Japanese Medical Students: Mixed Methods Study

**DOI:** 10.2196/84535

**Published:** 2026-01-14

**Authors:** Yuma Ota, Yoshikazu Asada, Saori Kubo, Takeshi Kanno, Machiko Saeki Yagi, Yasushi Matsuyama

**Affiliations:** 1 Medical Education Center Jichi Medical University Graduate School Shimotsuke Japan; 2 Medical Education Center Jichi Medical University Shimotsuke Japan; 3 Graduate School of Education Tohoku University Sendai Japan; 4 R & D Division of Career Education for Medical Professionals Medical Education Center Jichi Medical University Shimotsuke Japan; 5 Division of Gastroenterology Graduate School of Medicine Tohoku University Sendai Japan; 6 Medical Education Center Jichi Medical University Tochigi Japan

**Keywords:** artificial intelligence, undergraduate medical education, medical ethics, problem-based learning, mixed methods research, text mining

## Abstract

**Background:**

The rapid advancement of artificial intelligence (AI) has had a substantial impact on medicine, necessitating the integration of AI education into medical school curricula. However, such integration remains limited. A key challenge is the discrepancy between medical students’ positive perceptions of AI and their actual competencies, with research in Japan identifying specific gaps in the students’ competencies in understanding regulations and discussing ethical issues.

**Objective:**

This study evaluates the effectiveness of an educational program designed to improve medical students’ competencies in understanding legal and ethical AI-related issues. It addresses the following research questions: (1) Does this educational program improve students’ knowledge of AI and its legal and ethical issues, and what is each program element’s contribution to this knowledge? (2) How does this educational program qualitatively change medical students’ thoughts on these issues from an abstract understanding to a concrete and structured thought process?

**Methods:**

This mixed methods study used a single-group pretest and posttest framework involving 118 fourth-year medical students. The 1-day intervention comprised a lecture and problem-based learning (PBL) session centered on a clinical case. A 24-item multiple-choice questionnaire (MCQ) was administered at 3 time points (pretest, midtest, and posttest), and descriptive essays were collected before and after the intervention. Data were analyzed using linear mixed-effects models, the Wilcoxon signed-rank test, and text mining, including comparative frequency analysis and cooccurrence network analysis with Jaccard coefficients. An optional survey on student perceptions based on the attention, relevance, confidence, and satisfaction model was conducted (n=76, 64.4%).

**Results:**

Objective knowledge scores increased significantly from the pretest (median 17, IQR 15-18) to posttest (median 19, IQR 17-21; β=1.42; *P*<.001). No significant difference was observed between score gains during the lecture and PBL phases (*P*=.54). Qualitative text analysis revealed the significant transformation of cooccurrence network structures (Jaccard coefficients 0.116 and 0.121) from fragmented clusters to integrated networks. Students also used professional and ethical terminology more frequently. For instance, use of the term “bias” in patient explanations increased from 10 (8.5%) at pretest to 25 (21.2%) at posttest, while references to “personal information” in physician precautions increased from 36 (30.5%) to 50 (42.4%). The optional survey indicated that students’ confidence (mean 3.78, SD 0.87) was significantly lower than their perception of the program’s relevance (mean 4.20, SD 0.71; *P*<.001).

**Conclusions:**

This PBL-based program was associated with the improvements in knowledge and, more importantly, a structural transformation in students’ thinking about AI ethics from an abstract level to a concrete, clinically grounded reasoning. The discrepancy between quantitative and qualitative findings suggests limitations of MCQs in assessing higher-order skills fostered by PBL. Overall, this study indicates the potential of PBL as an effective pedagogical method for AI ethics education.

## Introduction

The rapid advancement of artificial intelligence (AI) has significantly affected the medical domain, which uses applications ranging from diagnostic support to treatment selection [1-3]. This necessitates the integration of AI education into medical school curricula to ensure that future physicians have the necessary competencies to use these technologies effectively and ethically [4-6]. Both educators and students recognize this necessity, and many studies support the implementation of systematic AI education [7,8]. However, despite the establishment of international recommendations, the integration of AI education into medical school curricula remains limited [3,6]. One of the key challenges in AI education identified by earlier research is the discrepancy between medical students’ perceptions of and competencies in AI [6,7,9]. Recent systematic reviews consistently indicate that despite generally maintaining a positive attitude toward AI’s health care potential, students have a moderate to low level of objective knowledge and practical skills [7]. This gap between the positive perceptions and insufficient competency acquisition of students indicates the need to integrate systematic and effective educational programs into the medical curriculum [9-11].

Furthermore, AI education poses not only technical challenges but also legal and ethical ones [1,2,12]. The use of AI can give rise to new legal and ethical issues, such as the risk of algorithmic bias resulting in patient discrimination, challenges related to patient privacy, and problems associated with accountability for AI-driven decisions [2,3,13]. Further, expert consensus studies indicate that nontechnical competencies such as ethics, law, communication, and collaboration are considered more important than advanced technical skills such as programming for physicians [8,11]. However, practical, case-based ethics education and standardized frameworks to teach these important topics are currently lacking [3,12,13].

This global context is highly relevant in Japan. In response to these trends, Japan’s Ministry of Education, Culture, Sports, Science, and Technology revised the national Model Core Curriculum for medical education in 2022, establishing “Competency in Utilizing Information Science and Technology” as a core competency for all future physicians [14,15]. This necessitated an investigation of the specific educational needs of Japanese medical students. A recent national cross-sectional study of sixth-year medical students in Japan clarified the acquisition status of specific competencies [16]. The study revealed particularly low self-assessment in three key learning objectives: (1) understanding the regulations, laws, and guidelines for information science and technology (IST) adoption in medicine; (2) discussing ethical issues, such as social disparities; and (3) understanding and discussing the potential applications of specific technologies, including AI, in medical care [16]. These results highlight the necessity of implementing focused educational interventions to address these gaps among Japanese medical students. To address these identified gaps, we chose a problem-based learning (PBL) framework for our educational intervention. This decision was directly informed by the findings of the aforementioned national study [16], which not only highlighted these specific areas of low confidence but also revealed a significant positive association between prior PBL experience and students’ self-assessed competence in these very domains. This suggests that the PBL methodology—which promotes active, case-based, and collaborative problem-solving—is a particularly effective approach for navigating the complex, nontechnical challenges of AI ethics and law.

Accordingly, this study aims to evaluate the effectiveness of an educational program designed to improve medical students’ competencies in addressing the legal and ethical issues associated with AI use. To this end, we developed and implemented a PBL program centered on a clinical case requiring fourth-year medical students to navigate AI-related ethical dilemmas. The use of the term “artificial intelligence” by Japan’s Model Core Curriculum highlights AI’s development period [14,15]. However, the educational program developed in this study considers recent technological advancements and the societal landscape to primarily address the ethical challenges posed by generative AI. Therefore, although this study uses the term AI in a broader sense in alignment with earlier research and the core curriculum, its content and examples mainly focus on generative AI. Finally, we address the following research questions (RQs):

RQ1: Does this educational program improve students’ knowledge of AI and its legal and ethical issues? Furthermore, which program elements improve knowledge, and how?

RQ2: How does the educational program qualitatively change medical students’ thoughts on the legal and ethical issues of AI? In particular, does their thinking shift from an abstract understanding to a more concrete and structured thought process that is based on the clinical context?

## Methods

### Study Design and Participants

This study adopted a mixed methods design using a single-group pretest and posttest framework to evaluate an educational intervention. Specifically, we used a convergent approach integrating quantitative assessment (multiple-choice questionnaire [MCQ] scores) and quantitative text analysis of qualitative data (student essays). The participants were fourth-year medical students (n=124) at Jichi Medical University who undertook the intervention program as a mandatory part of their curriculum during the 2025 academic year. The program was conducted immediately before the students commenced their full-time clinical clerkship; this time was selected because they were expected to soon encounter ethical and legal issues related to AI’s adoption in clinical practice.

### Educational Intervention

The intervention in this study was a 1-day (approximately 7 hours) program based on the PBL model [17]. The program’s learning objectives were selected such that they address the 3 key competency gaps (understanding regulations, discussing ethical issues, and understanding AI applications) that had low self-assessment scores among Japanese medical students in an earlier national cross-sectional study. The program required students to achieve the following three competencies based on a rubric proposed by earlier research [18]:

1. For standard clinical settings and cases, be able to take action based on regulations (laws, guidelines, etc) associated with IST’s use in medicine at a certain rate.
2. Be able to exemplify and discuss the ethical issues, such as the social disparities caused by the digital divide, potentially arising from the use of digital information and scientific technology in medicine and welfare.
3. Be able to understand the IST (AI) related to medicine and discuss it with the support of practitioners.

### PBL Case and Program Structure

At the beginning of the PBL-based program, a video depicting a challenging clinical scenario was shown. A patient who is diagnosed with “muscle pain” for his left shoulder pain by a medical AI consultation app is convinced of the diagnosis. However, his anxious son is concerned about the app’s reliability (since it lists acute myocardial infarction as the second possibility) and asks a medical student on clinical rotation to explain the AI diagnosis’ credibility. The case was designed to encompass multiple key AI ethics themes, including physician accountability, potential AI bias and safety issues, and empathetic communication.

The program was conducted over 1 complete day with the following structure (Table 1). First, at the beginning of the program, the learning objectives and the case video were presented. The case involved a scenario in which a medical student on clinical rotation was asked by a patient’s anxious son to explain the reliability of the medical AI consultation app that had been used to diagnose the patient’s left shoulder pain as muscle pain, a diagnosis the patient firmly believed. This case encompassed multiple key themes in AI ethics, including physician accountability, potential AI bias and safety issues, and empathetic communication. Subsequently, a preprogram assessment (pretest: an MCQ and descriptive questions) was conducted. Then, the foundational knowledge of AI was detailed in lecture 1, which referenced internationally acknowledged AI education content for medical students [3]. This lecture supported the acquisition of sufficient foundational knowledge to address the PBL case. It provided an overview of the fundamentals of AI, including its definition, strengths, machine and deep learning mechanisms, and the benefits and challenges of AI’s introduction into health care. Immediately after lecture 1, the second assessment (midtest: MCQ alone) was administered.

**Table 1:** Overview of the program components and schedule.

Content	Time	Activity format
Introduction: presentation of learning objectives and case	15 minutes	Individual
Assessment 1 (pretest): MCQ^a^ and descriptive questions	35 minutes	Individual
Lecture 1: foundational knowledge of AI^b^	35 minutes	Individual
Assessment 2 (midtest): MCQ	15 minutes	Individual
PBL^c^ session 1: confirmation of schedule and learning process	5 minutes	Group
PBL session 2: problem clarification	20 minutes	Group
PBL session 3: self-learning (including lunch break)	120 minutes	Individual
PBL session: problem-solving group work	70 minutes	Group
Lecture 2: case review and debriefing	40 minutes	Individual
Assessment 3 (posttest): MCQ and descriptive questions	35 minutes	Individual

^a^MCQ: multiple-choice questionnaire.

^b^AI: artificial intelligence.

^c^PBL: problem-based learning.

The PBL process proceeded in the following stages: (1) PBL learning process confirmation, (2) problem clarification, (3) self-directed learning (including a lunch break), and (4) a 70-minute problem-solving group work session. PBL was conducted in 16 small groups of 7-8 students each. The session comprised problem clarification, self-directed learning, and problem-solving phases and included a final 70-minute group work session where students used shared Google slides to collaboratively devise an explanation for the patient and his son. Five tutors facilitated the process using a standardized tutor guide and, to enhance discussion quality, key domestic AI guidelines [19,20] were distributed as group work materials.

At the end of the program, in lecture 2, an instructor reviewed the case to consolidate important concepts regarding AI’s legal and ethical issues. Immediately thereafter, the final assessment (posttest: an MCQ and descriptive questions) was administered. A detailed description of the PBL program’s development and theoretical underpinnings has been published [21].

### Data Collection

To evaluate the program’s effectiveness, we collected both quantitative and qualitative data using the university’s learning management system.

#### Using the MCQ

A 24-item MCQ, with its content aligned with lecture 1 materials, was used to assess students’ knowledge. Each item scored 1 point for a correct answer, the total possible score being 24 points. The questionnaire comprised 8 subscales, each with 3 items, covering the mechanisms of AI, the strengths of AI, machine learning, deep learning, overfitting, prompts, personal information protection, and challenges associated with AI use. To assess knowledge acquisition over time, the same set of questions was administered at 3 time points: before the intervention (pretest), after the lecture (midtest), and after the PBL program’s completion (posttest). To minimize recall bias, the order of the questions and their options was randomized at each administration.

#### Descriptive Essay Questions

Two descriptive essay questions were administered before the intervention (pretest) and after the program (posttest): “A patient literally believes the results diagnosed by a personal AI consultation tool. As a medical student, how will you explain the dangers of believing the results diagnosed by AI?” (question 1) and “When using AI as a physician, what points do you think require caution?” (question 2). These questions were designed to evaluate changes in students’ patient communication strategies and their understanding of the professional and ethical responsibilities associated with AI use.

#### Program Perception Survey

After the program, an optional survey was administered to assess students’ perceptions of the program based on the attention, relevance, confidence, and satisfaction (ARCS) model [22]. Students rated their agreement with statements for each component on a 5-point Likert scale (ranging from 1=strongly disagree to 5=strongly agree).

### Data Analysis

All statistical analyses were performed using R software (version 4.4.3; R Foundation for Statistical Computing) [23].

#### MCQ Data

The normality of data distribution for score gains was assessed using the Shapiro-Wilk test to determine the appropriate statistical test to compare educational phases. To assess the overall trajectory of the increase in score across the time points pretest, midtest, and posttest, a linear mixed-effects model was used. To compare the score gains between the lecture phase (midtest-pretest) and the PBL phase (posttest-midtest), a paired-samples 2 tailed *t* test was performed. Any violation of the assumption of normality was prespecified to result in the use of the nonparametric Wilcoxon signed-rank test as an alternative.

#### Descriptive Essay Data

To quantitatively and objectively analyze the content of free-text data, a quantitative text analysis (text mining) approach using several R packages was adopted. The analysis targeted the free-text responses to 2 questions at 2 time points: question 1 (patient explanation) at pretest and posttest and question 2 (physician precautions) at pretest and posttest. Further, data handling, aggregation, and visualization were performed using the *dplyr*, *tidyr*, *tidytext*, *widyr*, *igraph*, and *ggraph* R packages [24].

##### Morphological Analysis for Word Tokenization and Normalization

To segment Japanese free-text into analyzable word units, morphological analysis was performed using the *RMeCab* package (version 1.1.4) [25], which interfaces with the open-source engine MeCab, using the standard Information-technology Promotion Agency dictionary (ipadic). The analysis was limited to content words (nouns, verbs, and adjectives), with verbs and adjectives being normalized to nouns. Nouns alone are typically used as morphemes in network analysis [26].

##### Word Frequency Analysis

Extracted and normalized words were used to calculate the word frequencies for the responses to each question at each time point (question 1 at pretest, question 1 at posttest, question 2 at pretest, and question 2 at posttest). To assess the breadth of concept adoption across the cohort and mitigate the impact of outliers (eg, a single student repeating the same word), we treated word usage as a binary variable (presence or absence) for each student. Therefore, instead of raw counts, we calculated the number of students who used each word at least once to identify the most frequently used terms.

##### Comparative Word Frequency and Transition Analysis

To capture changes in descriptive content, the number of students using specific words was compared among time points for each question (pretest vs posttest for question 1 and pretest vs posttest for question 2). In addition to calculating the net difference in prevalence, we performed a transition analysis to examine individual-level changes. Specifically, we identified the number of students who newly adopted a word (nonuse at pretest to use at posttest) and those who dropped a word (use at pretest to nonuse at posttest). This allowed us to distinguish whether the observed changes in prevalence were driven by the acquisition of new concepts or the abandonment of previous ones.

##### Cooccurrence Network Analysis

To structurally clarify the contextual relationships among words irrespective of their frequencies, a quantitative cooccurrence network analysis was performed. In this study, cooccurrence involves capturing the semantic proximity of a pair of words appearing adjacently within the same sentence. Once text was segmented into sentence units, adjacent word pairs were extracted. Similar to the word frequency analysis, we calculated the number of students who used each word pair (binary counting) to avoid overrepresentation by single individuals, using the *widyr* package. Subsequently, a network diagram was created using the words mentioned by 5 or more students as nodes and their cooccurrence relationships as edges, with edge thickness indicating the relationship’s strength.

The Jaccard coefficient was used as a similarity metric to quantify the structural changes in these networks. The Jaccard coefficient measures the similarity between 2 sets by taking their intersection and dividing it by their union [26]. In this study, the coefficient was applied to compare entire network structures and was defined as the number of shared cooccurrence pairs (edges) divided by the total number of unique pairs across both pretest and posttest networks. We applied this metric because it assesses the number of common cooccurrence pairs without being affected by each pair’s frequency. Accordingly, we could determine the extent to which the network structure changed over time, which indicated a shift in conceptual relationships, rather than a simple change in discourse volume. We identified the numbers of common pairs (present at both time points) and unique pairs (present at only one time point), as well.

#### Program Perception Survey Data

For the optional ARCS survey data, the normality of the data distribution was assessed using the Shapiro-Wilk test. We elected to use parametric statistics by prioritizing the robustness of these tests given the adequate sample size, which mitigates the impact of potential deviations from normality. Descriptive statistics (mean and SD) were calculated for each of the 4 components. To clarify the differences between components, repeated-measures ANOVA was conducted. Further, pairwise *t* tests with a Bonferroni correction were used for post hoc analysis.

### Ethical Considerations

The study’s description stated that participation was voluntary and would not affect students’ grades. Only those medical students who agreed to participate accessed the questionnaire. The study was approved by the Ethics Review Committee of Jichi Medical University School of Medicine, Shimotsuke, Japan (approval number 24-137).

Informed consent was obtained electronically; students agreed to participate by accessing the questionnaire after reading the study description. To ensure privacy and confidentiality, all data were anonymized and stored securely with restricted access. No compensation was provided for participation.

## Results

### Participant Characteristics

In this study, a total of 118 fourth-year medical students participated. All participants provided complete data for both the MCQ and descriptive essay questions, which were the main aspects analyzed in this study. The participants were 71 (60.2%) male and 47 (39.8%) female students, all of whom were younger than 30 years.

### MCQ Scores: Descriptive Statistics and Overall Effectiveness

The MCQ’s internal consistency and reliability were assessed using the Cronbach α value at each time point. The α coefficients for the total score were 0.651 at pretest, 0.765 at midtest, and 0.782 at posttest. The α coefficients for the 8 subscales ranged from 0.631 to 0.760, indicating acceptable to good reliability. Table 2 depicts the detailed Cronbach α values for the total score and all subscales.

**Table 2:** Cronbach α coefficients for the multiple-choice questionnaire at each time point.

Factor	Pretest	Midtest	Posttest
Strengths of AI^a^	0.631	0.662	0.692
AI architecture	0.639	0.744	0.724
AI output issues	0.743	0.732	0.754
Prompts	0.657	0.707	0.696
Personal data leakage	0.76	0.708	0.77
Machine learning	0.666	0.751	0.726
Deep learning	0.704	0.729	0.757
Overfitting	0.716	0.733	0.743
Total score	0.651	0.765	0.782

^a^AI: artificial intelligence.

As per the Shapiro-Wilk test, the MCQ scores at none of the 3 time points were normally distributed (*P*<.001 for all values). Therefore, descriptive statistics were presented as median (IQR) values. The median total MCQ score increased across the time points, from 17 (IQR 15-18) at pretest to 18 (IQR 16-20) at midtest and 19 (IQR 17-21) at posttest (Table 3).

**Table 3:** Median (IQR) scores of the multiple-choice questionnaire (MCQ) at each time point. The total possible score range for the MCQ is 0-24.

Factor	Pretest, median (IQR)	Midtest, median (IQR)	Posttest, median (IQR)
Strengths of AI^a^	2 (2-3)	2 (2-3)	2 (2-3)
Mechanisms of AI	2 (2-3)	2 (2-3)	3 (2-3)
Challenges associated with AI use	2 (2-3)	2 (2-3)	2 (2-3)
Prompts	2 (1-2)	2 (1-3)	2 (1-3)
Personal information protection	3 (3-3)	3 (3-3)	3 (3-3)
Machine learning	2 (1-2)	2 (1-3)	2 (1-3)
Deep learning	1 (1-2)	2 (1-3)	2 (1-3)
Overfitting	2 (1-3)	2 (1-3)	2 (1-3)
Total score	17 (15-18)	18 (16-20)	19 (17-21)

^a^AI: artificial intelligence.

A linear mixed-effects model was used to assess the trajectory of this increase in scores. Results (Table 4) highlight the stepwise effect of the program. For instance, a statistically significant increase of 0.75 points from the baseline score at pretest was noted after the lecture phase at midtest (β=.75; *P*=.01). Furthermore, the end-of-program (posttest) score significantly exceeded the baseline score by 1.42 points (β=1.42; *P*<.001).

**Table 4:** Fixed effects from linear mixed‐effects model predicting total score. Model: Score ~ Time + (1 | ID); n=354 observations from 118 students.

Effect	Estimate β (SE)	*t* test (*df*)	*P* value
Intercept (pretest)	16.86 (0.34)	49.55 (179)	<.001
T2 (midtest)	0.75 (0.27)	2.79 (234)	.006
T3 (posttest)	1.42 (0.27)	5.30 (234)	<.001

To address the quantitative RQ1 regarding the PBL phase’s additional effect, the Wilcoxon signed-rank test was performed to compare score gains. The test found no statistically significant difference between the score gain during the lecture phase (midtest-Pretest) and that during the PBL phase (posttest-midtest) for either the total score (*V*=3084.5; *P*=.54) or any of the 8 subscales (Table 5).

**Table 5:** Wilcoxon signed-rank test comparing score gains between lecture (midtest-pretest) and PBL (posttest-midtest) phases.

Factor	*V* statistic	*P* value
Strengths of AI^a^	203.0	.058
Mechanisms of AI	1733.5	.21
Challenges associated with AI use	1347.0	.82
Prompts	1601.5	.76
Personal information protection	1044.0	.55
Machine learning	981.0	.61
Deep learning	120.0	.31
Overfitting	782.0	.90
Total score	3084.5	.54

^a^AI: artificial intelligence.

### Qualitative RQ

To address RQ2, free-text responses to question 1 (patient explanation) and question 2 (physician precautions) were analyzed by comparing word frequencies and cooccurrence network structures between pretest and posttests.

### Responses to Question 1

After the intervention, a significant increase in the number of students using words related to AI mechanisms, limitations, and applications was noted. In particular, a high number of students used “learning” (net increase of 18 students), “input” (+17), “bias” (+15), and “utilization” (+13). Transition analysis revealed that this increase was driven by new adoption; for instance, 37 (31.4%) students newly used the term “learning” and 20 (16.9%) students newly used “bias” in the posttest, indicating the acquisition of new perspectives. Contrastingly, the use of words describing the clinical case’s initial facts decreased. Only a few students used words such as “symptoms” (decrease of 11 students), “examination” (–11), and “explanation” (–9). Notably, transition analysis indicated that 23 (19.5%) students who used the term “symptoms” at pretest dropped it at posttest, suggesting a shift away from merely describing clinical manifestations. Table 6 depicts the top 10 words with the largest changes and their transition details.

**Table 6:** Changes in word usage frequencies and transition analysis for “patient explanation” (question 1; N=118).

Word			Pretest, n (%)		Posttest, n (%)		Net change		New adoption^a^, n (%)		Dropped^b^, n (%)	
**Words with increased usage**												
	Learning	32 (27.1)		50 (42.4)		+18		37 (31.4)		19 (16.1)		
	Input	20 (16.9)		37 (31.4)		+17		29 (24.6)		12 (10.2)		
	Bias	10 (8.5)		25 (21.2)		+15		20 (16.9)		5 (4.2)		
	Utilization	12 (10.2)		25 (21.2)		+13		20 (16.9)		7 (5.9)		
	Misdiagnosis	7 (5.9)		20 (16.9)		+13		15 (12.7)		2 (1.7)		
	Information	24 (20.3)		35 (29.7)		+11		25 (21.2)		14 (11.9)		
	Case	31 (26.3)		40 (33.9)		+9		22 (18.6)		13 (11)		
	Hospital	9 (7.6)		17 (14.4)		+8		13 (11)		5 (4.2)		
	Convenient	4 (3.4)		10 (8.5)		+6		9 (7.6)		3 (2.5)		
	Judgment	32 (27.1)		38 (32.2)		+6		16 (13.6)		10 (8.5)		
**Words with decreased usage**												
	Symptoms	27 (22.9)		15 (12.7)		–12		11 (9.3)		23 (19.5)		
	Examination	18 (15.3)		7 (5.9)		–11		4 (3.4)		15 (12.7)		
	Explanation	22 (18.6)		13 (11)		–9		8 (6.8)		17 (14.4)		
	Presentation	12 (10.2)		4 (3.4)		–8		2 (1.7)		10 (8.5)		
	During	11 (9.3)		4 (3.4)		–7		2 (1.7)		9 (7.6)		
	Consultation	23 (19.5)		16 (13.6)		–7		8 (6.8)		15 (12.7)		
	View	8 (6.8)		2 (1.7)		–6		1 (0.8)		7 (5.9)		
	Result	29 (24.6)		24 (20.3)		–5		13 (11)		18 (15.3)		
	Certain	23 (19.5)		18 (15.3)		–5		8 (6.8)		13 (11)		
	Many	15 (12.7)		10 (8.5)		–5		5 (4.2)		10 (8.5)		

^a^The term “new adoption” indicates students who did not use the word at pretest but used it at posttest, reflecting the acquisition of new concepts.

^b^“Dropped” indicates students who used the word at pretest but excluded it at posttest.

**Figure 1:**
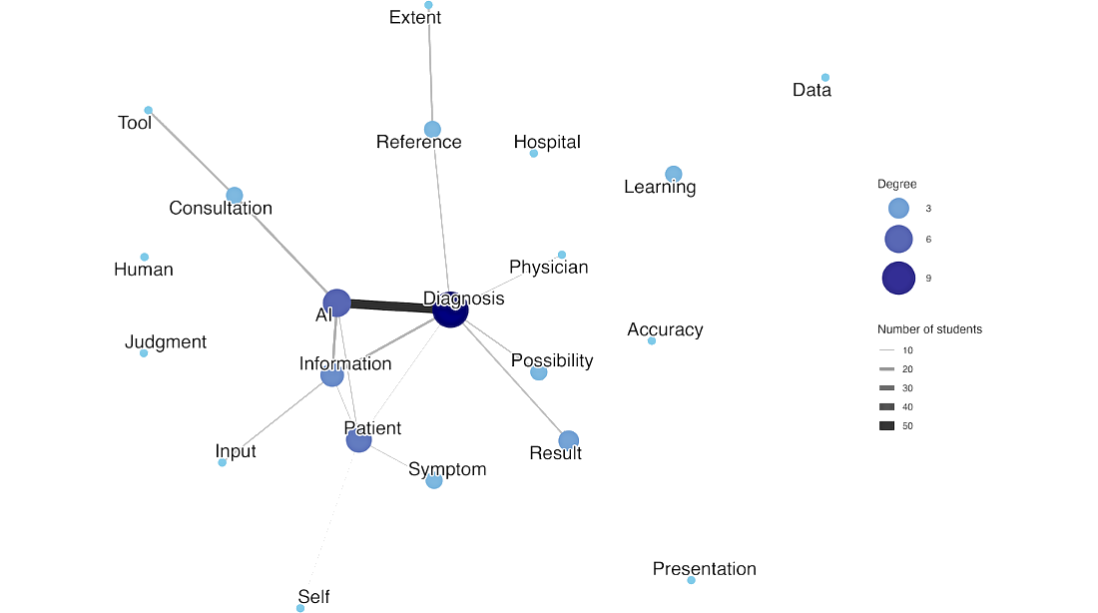
The network visualizes the relationships between keywords in students’ explanations to the patient before the program. Each node represents a word, and the size and color of the node indicate its degree (number of connections). The thickness and color of the edge (line) connecting 2 nodes represent the number of students who used those 2 words adjacently.

**Figure 2:**
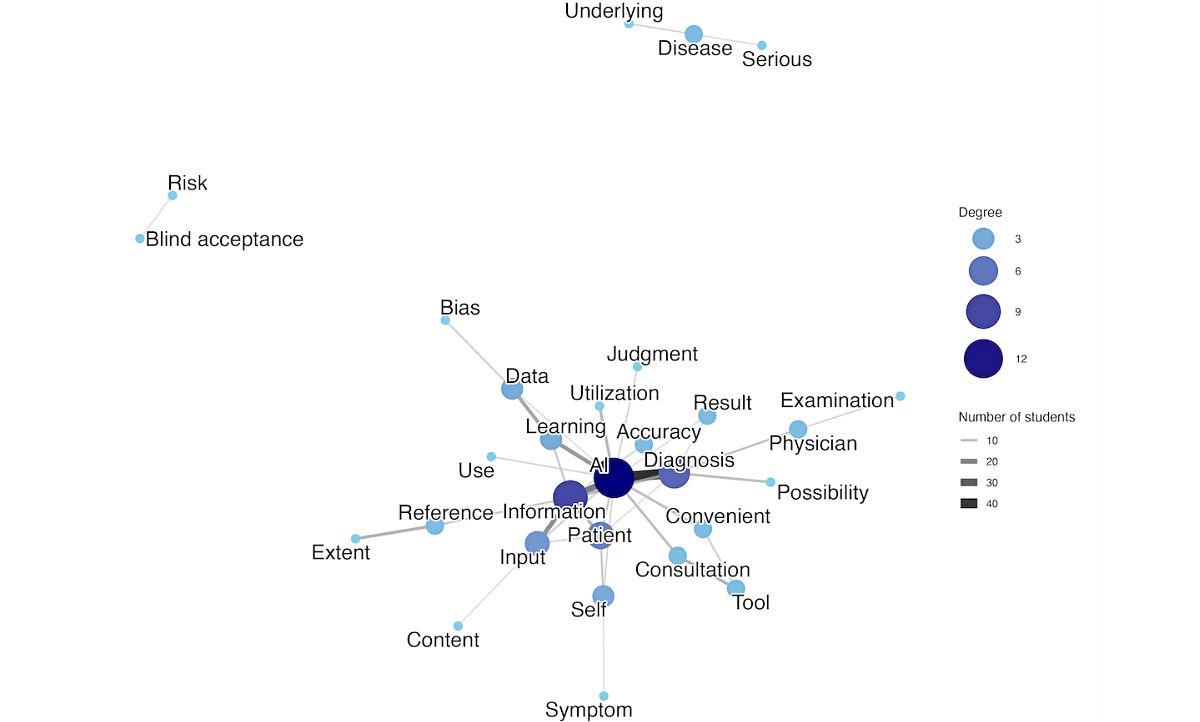
The network visualizes the relationships between keywords in students’ explanations to the patient after the program.

Subsequently, we assessed the structural changes in students’ thinking by comparing the cooccurrence networks between pretest and posttests. The patient explanation network’s Jaccard coefficient was 0.1156, indicating very low similarity between the 2 time points. This dramatic shift was driven by a large number of unique pairs at each time point. The network had only 264 shared cooccurrence pairs; however, 1030 unique pairs existed exclusively at pretest, and 989 unique pairs emerged at posttest. These quantitative findings are supported by a qualitative analysis of the network structures. In the pretest, the network was structured around the case’s core elements (Figure 1). However, the network became highly complex and specific at posttest, with keywords like “learning” and “data” being directly linked to “AI” and new vocabulary, such as “bias” and “blind acceptance,” explaining the limitations and risks of AI use (Figure 2).

### Responses to Question 2

For question 2, a significant increase in the use of words related to professional and ethical responsibilities was observed. The number of students using key concepts such as “information” (net increase of 25 students), “personal info” (+14), “accuracy” (+13), and “bias” (+10) increased significantly, as well. Notably, 31 (26.3%) students newly adopted the term “personal info” in their posttest responses, suggesting a shift in their awareness of privacy issues. Contrastingly, the number of students using abstract or cautionary words, such as “possibility” (decrease of 9 students), “reference” (–5), and “keep in mind” (–5), decreased. Notably, transition analysis revealed that 30 (25.4%) students dropped the term “reference” and 24 (20.3%) students dropped “possibility” in their posttest responses, suggesting a departure from vague expressions. Table 7 depicts the top 10 words with the largest changes and their transition details.

**Table 7:** Changes in word usage frequencies and transition analysis for “physician precautions” (question 2; N=118).

Word		Pretest, n (%)	Posttest, n (%)	Net change	New adoption^a^, n (%)	Dropped^b^, n (%)
**Words with increased usage**						
	Information	56 (47.5)	81 (68.6)	+25	38 (32.2)	13 (11)
	AI^c^	49 (41.5)	66 (55.9)	+17	29 (24.6)	12 (10.2)
	Personal info	36 (30.5)	50 (42.4)	+14	31 (26.3)	17 (14.4)
	Accuracy	22 (18.6)	35 (29.7)	+13	26 (22)	13 (11)
	Input	2 (1.7)	12 (10.2)	+10	12 (10.2)	2 (1.7)
	Bias	5 (4.2)	15 (12.7)	+10	13 (11)	3 (2.5)
	Patient	18 (15.3)	26 (22)	+8	17 (14.4)	9 (7.6)
	Assistance	11 (9.3)	18 (15.3)	+7	12 (10.2)	5 (4.2)
	Final	14 (11.9)	20 (16.9)	+6	15 (12.7)	9 (7.6)
	View	0 (0)	5 (4.2)	+5	5 (4.2)	0 (0)
**Words with decreased usage**						
	Possibility	37 (31.4)	28 (23.7)	–9	15 (12.7)	24 (20.3)
	Reference	67 (56.8)	61 (51.7)	–6	24 (20.3)	30 (25.4)
	Oneself	17 (14.4)	11 (9.3)	–6	4 (3.4)	10 (8.5)
	Keep in mind	15 (12.7)	10 (8.5)	–5	7 (5.9)	12 (10.2)
	Presentation	12 (10.2)	7 (5.9)	–5	5 (4.2)	10 (8.5)
	Knowledge	15 (12.7)	10 (8.5)	–5	5 (4.2)	10 (8.5)
	Examination	8 (6.8)	4 (3.4)	–4	3 (2.5)	7 (5.9)
	Caution	54 (45.8)	50 (42.4)	–4	24 (20.3)	28 (23.7)
	Output	5 (4.2)	1 (0.8)	–4	1 (0.8)	5 (4.2)
	Source	17 (14.4)	13 (11)	–4	6 (5.1)	10 (8.5)

^a^The term “new adoption” indicates students who did not use the word at pretest but used it at posttest, reflecting the acquisition of new concepts.

^b^“Dropped” indicates students who used the word at pretest but excluded it at posttest.

^c^AI: artificial intelligence.

**Figure 3:**
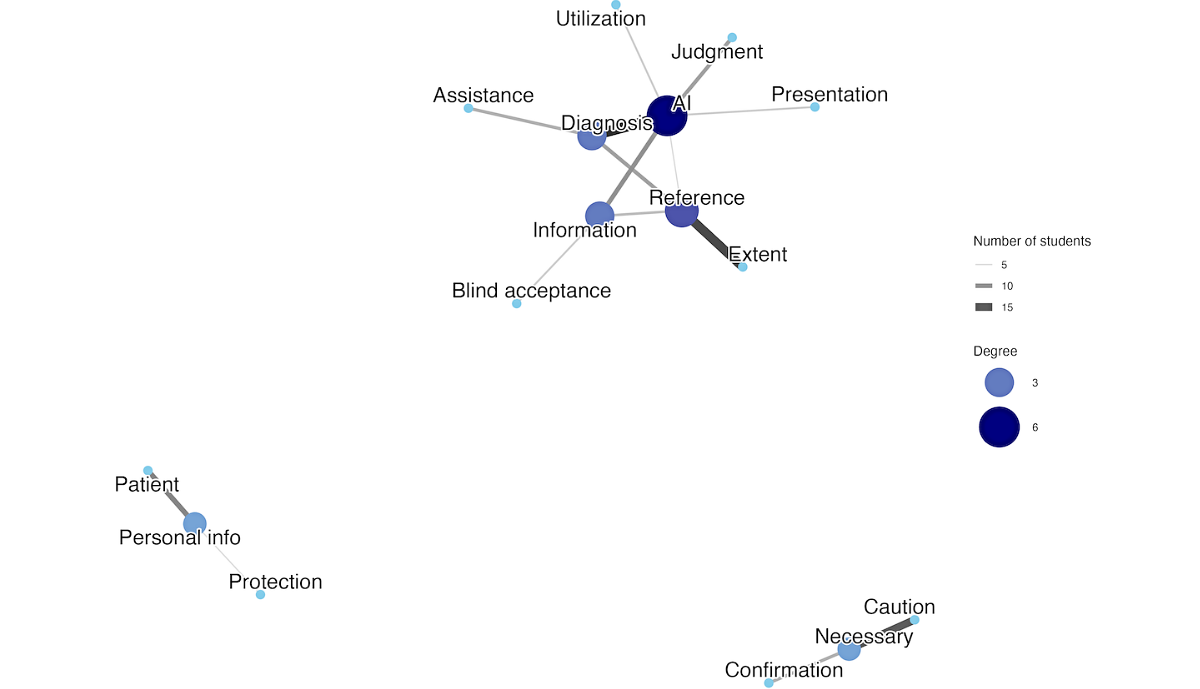
The network visualizes the relationships between keywords regarding precautions for physicians before the program.

**Figure 4:**
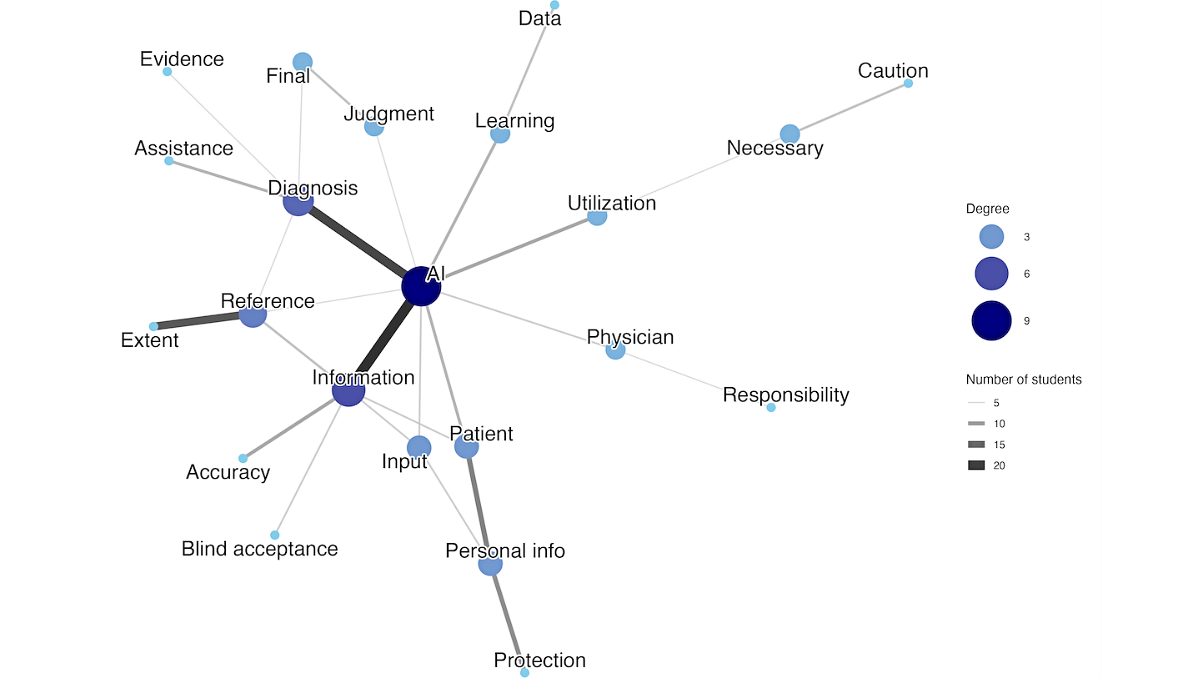
The network visualizes the relationships between keywords regarding precautions for physicians after the program.

The cooccurrence network for physician precautions showed a significant change, as well. The Jaccard coefficient was 0.1213, confirming the occurrence of a major structural transformation. The low score was driven by a large number of unique pairs. Only 191 pairs were shared, whereas 631 unique pairs were found exclusively at pretest, and 753 new pairs appeared at posttest. The higher number of unique pairs found at posttest compared to pretest suggests that students not only abandoned previously acquired cautionary topics but also actively explored a wider range of new professional and ethical concerns. These quantitative results were corroborated by a qualitative analysis of network structures. The pretest network comprised 3 separate, unconnected clusters (Figure 3), which transformed into a single, integrated network at posttest, where “AI” and “information” acted as hubs (Figure 4).

### Students’ Perceptions of the Program

Among the 118 participants, 76 (64.4%) completed an optional postprogram survey based on the ARCS model. On a 5-point Likert scale, students rated the program highly for attention (mean 4.01, SD 0.62), relevance (mean 4.20, SD 0.71), confidence (mean 3.78, SD 0.87), and satisfaction (mean 4.03, SD 0.75). A repeated-measures ANOVA was conducted to compare and reveal a statistically significant difference between the mean scores of the 4 ARCS components (*F*_3, 225_=13.15; *P*<.001). Additionally, post hoc tests with Bonferroni correction revealed that the mean score for confidence was significantly lower than the scores for attention, relevance, and satisfaction. Furthermore, relevance was rated significantly higher than satisfaction.

## Discussion

### Principal Findings

This study’s primary objective was to evaluate the effectiveness of a newly developed PBL program. Further, this study addresses the following RQs:

RQ1: The educational program improves students’ objective knowledge of AI. Accordingly, we hypothesized that foundational knowledge would increase after the lecture phase (from pretest to midtest) and the subsequent PBL phase (from midtest to posttest) would produce a significant additional increase in practical and ethical knowledge.

RQ2: The educational program qualitatively transforms students’ thinking regarding AI-related ethical challenges from an abstract understanding to a concrete and structured thought process based on the clinical context.

Regarding RQ1, although a significant improvement in MCQ scores was observed for the program in its entirety, no statistically significant “additional effect” of the PBL phase was observed. Regarding RQ2, text mining results suggested that after the intervention, students’ thinking underwent a qualitative transformation, which was characterized by the use of specific and professional terms, such as “bias,” “personal information,” and “guidelines,” and the development of a structured thought process.

### Interpretation of Findings

Regarding RQ1, a clear additional effect of PBL was not detected in MCQ scores due to 2 main reasons. First, the knowledge measured by MCQ was the foundational content that probably had been efficiently acquired during the lecture phase. The second reason is the limitation of the MCQ format itself. As argued by van der Vleuten and Schuwirth [27] in their paper on PBL assessment, traditional standardized knowledge tests cannot adequately assess abilities such as clinical reasoning and metacognition, which are fostered by PBL. Consequently, the lack of significant MCQ score improvement during the PBL phase does not imply a lack of learning; rather, it suggests that the unique contribution of PBL lies in the qualitative structural transformation of students’ thinking—specifically, a shift from abstract knowledge to context-based application—which is difficult to capture with standard testing. Although MCQs are suitable to measure objective knowledge, they may have low sensitivity in capturing changes in the higher-order practical skills fostered by PBL. Indeed, reviews of earlier research on PBL’s effectiveness indicate that whereas knowledge acquisition is comparable to or better than traditional lecture-based learning, PBL is more effective in improving higher-order skills, such as communication, problem-solving, self-learning, and metacognitive competencies [28,29]. Therefore, the reason a statistically significant additional effect of PBL was not detected in MCQ scores is that PBL’s true effect was reflected in the qualitative changes in students’ written responses, rather than the multiple-choice questions themselves.

This is clarified by the results for RQ2. The quantitative text analysis revealed that the program structurally transformed students’ thinking. Among the responses to question 1 (patient explanation), the increased use of words related to AI’s mechanisms and limitations was driven by new adoption. For instance, 37 (31.4%) students newly adopted the term “learning” and 20 (16.9%) newly adopted “bias.” This suggests that students based their explanations of the bases and limitations of AI’s judgments on the transparency principle (explainability) and accountability principle detailed in the AI Utilization Guideline and AI Business Guideline [19,20]. Conversely, 23 (19.5%) students dropped the term “symptoms,” indicating a shift away from merely describing clinical manifestations to addressing the underlying logic of the AI. Similarly, among the responses to question 2 (physician precautions), the significant increase in the use of words related to ethical and professional responsibilities was also driven by new adoption. Notably, 31 (26.3%) students newly adopted “personal info,” which can be interpreted as the result of students’ efforts to learn and apply the privacy principle [19,20]. Simultaneously, we observed a decrease in abstract vocabulary. Transition analysis revealed that 30 (25.4%) students dropped the term “reference” and 24 (20.3%) dropped “possibility.” This reduction in vague or passive expressions suggests that students adopted a more specific and responsible professional stance. The cooccurrence network analysis’ quantitative results support the inference that the qualitative change in thinking was accompanied by the structuring of knowledge. The cooccurrence network for question 1 revealed a very low Jaccard coefficient (0.1156) between the pretest and postintervention networks. This indicates the significant transformation of the contextual relationships between words. For 264 common pairs, there were 1030 unique pairs before and 989 unique pairs after the intervention; accordingly, new knowledge and concepts were established by replacing traditional knowledge. Moreover, the Jaccard coefficient for the cooccurrence network of question 2 was low (0.1213), indicating a similar structural change. In the question 2 cooccurrence network, whereas “AI’s function,” “privacy,” and “general caution” existed as separate clusters at pretest, a more integrated network was formed with the word “information” as a new hub, connecting “AI” and “diagnosis” with important concepts like “accuracy,” at posttest. The study’s quantitative and qualitative findings resonate with the argument of McCoy et al [12] that medical students must possess knowledge about AI. They argue that future physicians must be able to not only “use” AI but also “interpret” its results, recognize potential errors and biases, and “explain” the results and processes to patients and other health care professionals. In this study, the qualitative improvement in the postintervention descriptions highlight that students’ abilities expanded from the “use” perspective to the “interpret and explain” perspective.

One reason for fostering this qualitative transformation of thinking is interpreted from the perspective of the 4 components of the ARCS model of motivation. First, the clinical case presented as a video at the beginning of the group work session effectively captured students’ attention (mean rating 4.01, SD 0.62). A systematic review of emotions’ role in medical education highlights the significance of emotional experiences in the learning process [30]. The video depicted a medical student facing an ethical dilemma and potentially fostered emotional engagement, thereby enhancing the students’ motivation for subsequent learning. Second, their awareness of the challenge involved in responding to a patient who is overreliant on AI probably enabled students to clearly understand the learning content’s relevance to their future practice (mean rating 4.20, SD 0.71). This educational design aligns with the principles of “authentic learning,” which involves the solving of complex, real-world contextual problems to enhance learners’ competency in the practical application of knowledge [31]. Further, students perceived the case in this program as a personal and relevant task due to the effective functioning of the elements of authentic learning. On the other hand, the students’ confidence in addressing a complex ethical issue resulted in a mean rating of 3.78 (SD 0.87), which was significantly lower than the values for attention, relevance, and satisfaction (*F*_3, 225_=13.15; *P*<.001). Therefore, when the program prompted a qualitative transformation in the students’ thinking, a single intervention was insufficient for them to acquire complete confidence in such a complex and uncertain topic as AI ethics. This program was limited to considering the explanation’s content to the patient and his son and did not provide any opportunities for practical application, such as a role-play of the explanation. This lack of practical experience probably contributed to the students’ low confidence in applying knowledge. An earlier study highlights the importance of maintaining a balance between confidence and humility and adopting a “not-knowing” stance as a key clinical leadership competency aligned with the Japanese health care culture [31]. The low confidence scores observed in our study were interpreted as not any failure to learn, but rather the development of the intellectual humility required by a professional, particularly those confronting complex and uncertain topics such as AI ethics.

However, the high ratings for components other than confidence, particularly attention and relevance, comprised a strong motivation for students to clearly understand the subject’s importance and engage in self-directed learning [21]. The effective functioning of these elements enabled the students to complete a learning process involving deep thought, and the resulting insights probably led to the final Satisfaction (mean rating 4.03, SD 0.75) and structuring of thought observed in this study.

### Research Significance and Limitations and Future Research Scope

The primary significance of this study is that it develops, implements, and comprehensively evaluates an educational program themed on a realistic and novel clinical challenge that may be faced by physicians in this era of widespread AI use: responding to a patient who relies heavily on AI. Presenting this ethical dilemma as a video teaching material has pedagogical validity because a systematic literature review indicates that video materials can effectively stimulate student discussion in ethics education [32]. Furthermore, the visualization of the program’s educational effect as not only a change in objective knowledge but also a qualitative and structural change in thinking using text mining is a novel approach to evaluate the effects of constructivist learning on complex topics such as AI ethics.

This study has several limitations. First, this study used a single-group pretest and posttest design without a control group. Therefore, while we observed significant changes, we cannot fully rule out the influence of external factors or natural maturation. Second, because it was conducted at a single university in Japan, the generalizability of its results may be limited by the specific cultural and educational context. However, we emphasize that the core framework of this program—integrating technical AI knowledge with ethical PBL scenarios—is adaptable. Future multiinstitutional collaborative studies are needed to validate whether similar structural changes in thinking occur in different educational and cultural contexts. Third, this study considered only the program’s short-term effects on learning. Therefore, the participating students must be subjected to a follow-up study when they proceed to their clinical clerkships to verify the learning effects’ persistence. Clarifying how the qualitative transformation in thinking indicated by this study influences students’ decision-making and behavior in clinical settings is important to evaluate the value of this type of educational programs. Fourth, as mentioned earlier, the MCQ format may not fully capture the effects of PBL. Therefore, there is an urgent need to develop and validate new assessment measures that can objectively assess the practical reasoning and communication skills that cannot be captured by applying MCQs alone.

### Conclusions

The results of this study suggest that the PBL program developed and implemented in this study influenced both medical students’ knowledge of AI and their thoughts on its ethical challenges. Although the program in its entirety resulted in a significant increase in objective knowledge, a statistically significant additional effect was not confirmed from the PBL phase alone. However, there was a clear change in the qualitative data revealed by text mining: Students’ thinking underwent qualitative transformation from an abstract level to a concrete and structured thought process based on the clinical context. Therefore, this study indicates the potential of PBL as an effective pedagogical method to foster higher-order thinking skills in complex topics, such as AI ethics. Hence, this program’s design and evaluation framework can serve as a practical model to develop and evaluate future AI-related curricula in medical education.

## Abbreviations

AI: artificial intelligence
ARCS: attention, relevance, confidence, and satisfaction
IST: information science and technology
MCQ: multiple-choice questionnaire
PBL: problem-based learning
RQ: research question

## Data Availability

The datasets generated and/or analyzed during this study are not publicly available due to ethical constraints but are available from the corresponding author on reasonable request.
